# Supplementary material for: The Cabrières Biota (France) provides insights into Ordovician polar ecosystems
Source: Nat Ecol Evol. 2024 Feb 9;8(4):651–62. doi: 10.1038/s41559-024-02331-w (PMC11009115; doi:10.1038/s41559-024-02331-w)
Supplement: Supplementary file 1 — Reporting Summary [file 41559_2024_2331_MOESM1_ESM.pdf]

nature portfolio

Corresponding author(s): Farid Saleh

Last updated by author(s): Dec 26, 2023

Reporting Summary

Nature Portfolio wishes to improve the reproducibility of the work that we publish. This form provides structure for consistency and transparency in reporting. For further information on Nature Portfolio policies, see our Editorial Policies and the Editorial Policy Checklist.

Statistics

For all statistical analyses, confirm that the following items are present in the figure legend, table legend, main text, or Methods section.

n/a	Confirmed	
☒	☐	The exact sample size (*n*) for each experimental group/condition, given as a discrete number and unit of measurement
☒	☐	A statement on whether measurements were taken from distinct samples or whether the same sample was measured repeatedly
☒	☐	The statistical test(s) used AND whether they are one- or two-sided<br>*Only common tests should be described solely by name; describe more complex techniques in the Methods section.*
☒	☐	A description of all covariates tested
☒	☐	A description of any assumptions or corrections, such as tests of normality and adjustment for multiple comparisons
☒	☐	A full description of the statistical parameters including central tendency (e.g. means) or other basic estimates (e.g. regression coefficient) AND variation (e.g. standard deviation) or associated estimates of uncertainty (e.g. confidence intervals)
☒	☐	For null hypothesis testing, the test statistic (e.g. *F*, *t*, *r*) with confidence intervals, effect sizes, degrees of freedom and *P* value noted<br>*Give P values as exact values whenever suitable.*
☒	☐	For Bayesian analysis, information on the choice of priors and Markov chain Monte Carlo settings
☒	☐	For hierarchical and complex designs, identification of the appropriate level for tests and full reporting of outcomes
☒	☐	Estimates of effect sizes (e.g. Cohen's *d*, Pearson's *r*), indicating how they were calculated

Our web collection on statistics for biologists contains articles on many of the points above.

Software and code

Policy information about availability of computer code

Data collection	The following softwares were used during electron microscopy, mutispectral imaging, and optical photographs data collections: Helicon Focus software, ImageJ, Bruker Nano Analytics and PyMCA.
Data analysis	No software or code was used for data analysis.

For manuscripts utilizing custom algorithms or software that are central to the research but not yet described in published literature, software must be made available to editors and reviewers. We strongly encourage code deposition in a community repository (e.g. GitHub). See the Nature Portfolio guidelines for submitting code & software for further information.

Data

Policy information about availability of data

All manuscripts must include a data availability statement. This statement should provide the following information, where applicable:

- Accession codes, unique identifiers, or web links for publicly available datasets
- A description of any restrictions on data availability
- For clinical datasets or third party data, please ensure that the statement adheres to our policy

All the data needed to reproduce this manuscript are available in the Main Text and the Extended Data Figures. All material can be accessed at the public collections of the University of Lyon1 (France). The collections are public with no access restrictions. All necessary information have been deposited with the specimens at the collections.

Research involving human participants, their data, or biological material

Policy information about studies with human participants or human data. See also policy information about sex, gender (identity/presentation), and sexual orientation and race, ethnicity and racism.

Reporting on sex and gender	n/a
Reporting on race, ethnicity, or other socially relevant groupings	n/a
Population characteristics	n/a
Recruitment	n/a
Ethics oversight	n/a

Note that full information on the approval of the study protocol must also be provided in the manuscript.

Field-specific reporting

Please select the one below that is the best fit for your research. If you are not sure, read the appropriate sections before making your selection.

☐ Life sciences ☐ Behavioural & social sciences ☒ Ecological, evolutionary & environmental sciences

For a reference copy of the document with all sections, see nature.com/documents/nr-reporting-summary-flat.pdf

Ecological, evolutionary & environmental sciences study design

All studies must disclose on these points even when the disclosure is negative.

Study description	This study describes new fossil assemblage from the Early Ordovician of France
Research sample	Fossils found at outcrops were described to give a view of the general biota
Sampling strategy	Palaeontological excavation of an outcrop using classical tools such as hammers
Data collection	The authors collected all the fossils found at outcrop, and inferred their taxonomic position
Timing and spatial scale	Fieldwork happened over the past 5 years whenever weather permitted, with a total time spent on outcrop of over 50 days. Fossils come from a single quarry with a 1m thickness
Data exclusions	No data is excluded as all recognizable fossils were collected from the outcrop
Reproducibility	The fossils are deposited in a public museum and can be accessed by anyone who wishes to restudy these
Randomization	n/a
Blinding	n/a

Did the study involve field work? ☒ Yes ☐ No

Field work, collection and transport

Field conditions	Fieldwork happened in spring and summer
Location	Cabrières Village Southern France, details are provided in the manuscript text, and with the fossils in the collections
Access & import/export	All data collection happened in accordance with local and French law. All fossils are housed in a public institution in France, their country of origin.
Disturbance	Only small tools, like hammers, were used to excavate the site. No major disturbances to surrounding fauna or flora happened when excavating the fossils.

Reporting for specific materials, systems and methods

We require information from authors about some types of materials, experimental systems and methods used in many studies. Here, indicate whether each material, system or method listed is relevant to your study. If you are not sure if a list item applies to your research, read the appropriate section before selecting a response.

Materials & experimental systems

n/a	Involved in the study
☒	☐ Antibodies
☒	☐ Eukaryotic cell lines
☐	☒ Palaeontology and archaeology
☒	☐ Animals and other organisms
☒	☐ Clinical data
☒	☐ Dual use research of concern
☒	☐ Plants

Methods

n/a	Involved in the study
☒	☐ ChIP-seq
☒	☐ Flow cytometry
☒	☐ MRI-based neuroimaging

Palaeontology and Archaeology

Specimen provenance	From outcrops near the Cabrières Village in France. No special permit is needed for this site at the moment. However, fieldwork permit were obtained from the University of Lyon1 when researchers visited the site. Also all discovered fossils are permanently housed in their country of origin, France.
Specimen deposition	Specimens are deposited in the public collections of the University Claude Bernard, Lyon1 in France
Dating methods	Widely accepted graptolite and trilobite biozones dating the outcrop to the Early Ordovician

☒ Tick this box to confirm that the raw and calibrated dates are available in the paper or in Supplementary Information.

Ethics oversight	No ethical approval was needed.

Note that full information on the approval of the study protocol must also be provided in the manuscript.

Plants

Seed stocks	n/a
Novel plant genotypes	n/a
Authentication	n/a
